# The Supervision of Hospital Alterations

**Published:** 1910-05-07

**Authors:** 


### THE SUPERVISION OF HOSPITAL ALTERATIONS.

The *Carpenter and Builder* remarks, in an encouraging sort of way, that a can is of no use for the position of clerk of works unless he has full practical knowledge of all the modern handicrafts concerned in the construction of a large building. Unless, that is (and it sounds plausible), he is a judge of stone, brick, and timber; can say how long the masons are entitled to keep the joiners, and the joiners the decorators, waiting; knows how the "pitching" of a road is done, and whether or not paint is mixed as it should be; has a graspon the relative advantages of concentric and parallel wiring—unless all such things are well within his capacity, he is not worth his wages. And, indeed, no more is he when the disadvantages of the situation are taken into account. For let this official be as competent and conscientious as he will, he cannot escape the consequences of the fact that he is not the employer of those under his supervision or a person of great influence with the man who is. As a rule he has no pretensions to an appearance of gentility, which (regrettably) has still in this country so much influence over the class from which artisans are drawn. Further, a fact more to be deplored, he is bound hand and foot by that code of morality which, if not extending to commercial affairs in general, certainly characterises the behaviour of three out of every five contractors. Lastly, his connection with the building ceases when it is finished.

A question which should therefore interest hospital secretaries, medical superintendents, and leisured committeemen in search of a useful hobby, is whether, given an individual free of these disabilities, and possessed of an impressive personality, small additions and all sorts of repaiis in connection with an institution cannot be done under home management rather better, and much more cheaply than if put out to contract. The reference is more particularlw to institutions which, from their large size or remoteness from towns, employ constantly the nucleus of the staff required.

It has been said that the question may be interesting, and indeed the word enjoyable might have been substituted. First, there is the satisfaction proceeding from discharge of surplus potential energy, and from the outraging of the already mentioned code of morality—the notion that charitable institutions, like railway companies, "stand to be shot at," and that to "best" them is about as venial as stealing matches from the smoking-room of one's golf club. Personal kudos constitutes another motive, of course, but a stronger one, to anybody fond of observation, is the glimpses obtainable into certain mental characteristics of what Anglo-Indians term the subordinate class. For although the first thing is to secure the services of good, experienced workmen, yet it is every whit as important to realise the freemasonry that exists among artisans and get some inkling of their true attitude to the educated man. Navvies, carpenters, electricians, and engineers, diverse as their occupations seem to be, have nevertheless much knowledge in common which they well know the "gentleman" does not possess. They think very little, therefore, of his opinion in small practical details. But, on the other hand, they distrust their own judgment in matters of taste. Conservative and rigid as the British working man's methods are, he becomes adaptable when told by a superior that such and such an arrangement will "look better." It flatters him to be seen in consultation with a gentleman foreman, who, moreover, does not get up at an ungodly hour in the morning to exact strict punctuality. If this individual can make it agreeable to his own personal dignity to show sympathy, geniality, and *esprit de corps*—occasionally even to work side by side with his men—their hearts will be won at once. All the little delinquencies and mutual connivances which employés are accustomed to justify to themselves by class envy and a feeling of solidarity—all these troubles largely disappear, to be replaced by an amiable emulation in accomplishing well the common purpose.

And this makes all the difference in the world when it comes to results. Just as the nurse, with the opportunities of close observation which her menial attendance on the patient gives her, may have good reason for a quiet distrust of the doctor's prognosis, so the workmen on many a building may be laughing in their sleeves at the scene of universal congratulation which marks the opening ceremony—remembering, perhaps, paint that is all paraffin or concrete that is all sand, conscious of a disconnected soil pipe or two silently creating a dangerous nuisance a foot underground, feeling it to be in their power to point to half a dozen spots where good surplus material was buried just to save the trouble of collecting it.

It is not argued that dispensing with a middleman is all plain sailing. Some of the information proffered by subordinates needs a good deal of sifting, for few artisans have accomplished what Dr. Johnson declares every wise man manages to effect, and that is self-cure of the itch for astonishing people. But a little commonsense, and a little study of the manuals which nowadays are written about every subject under the sun, will provide against this danger. If the plan outlined above be tried on an appropriate (not too large) scale by someone who finds it no trouble, work can be turned out of a quality only to be provided by firms whose services command a price beyond the pocket of most hospital committees.

